# Enhancement of Salinity Stress Tolerance in Lettuce (*Lactuca sativa* L.) via Foliar Application of Nitric Oxide

**DOI:** 10.3390/plants12051115

**Published:** 2023-03-01

**Authors:** Hasan Sardar, Zubair Khalid, Muhammad Ahsan, Safina Naz, Aamir Nawaz, Riaz Ahmad, Kashif Razzaq, Saikh M. Wabaidur, Cédric Jacquard, Ivan Širić, Pankaj Kumar, Sami Abou Fayssal

**Affiliations:** 1 Department of Horticulture, Faculty of Agricultural Sciences and Technology, Bahauddin Zakariya University, Multan 60800, Pakistan; 2 Department of Horticultural Sciences, The Islamia University of Bahawalpur, Bahawalpur 63100, Pakistan; 3 Department of Horticulture, The University of Agriculture, Dera Ismail Khan 29111, Pakistan; 4 Department of Horticulture, Muhammad Nawaz Shareef University of Agriculture, Multan 60000, Pakistan; 5 Department of Chemistry, College of Science, King Saud University, Riyadh 11451, Saudi Arabia; 6 Research Unit Induced Resistance and Plant Bioprotection, University of Reims, EA 4707 USC INRAe 1488, SFR Condorcet FR CNRS 3417, 51100 Reims, France; 7 University of Zagreb, Faculty of Agriculture, Svetosimunska 25, 10000 Zagreb, Croatia; 8 Agro-Ecology and Pollution Research Laboratory, Department of Zoology and Environmental Science, Gurukula Kangri (Deemed to Be University), Haridwar 249404, India; 9 Department of Agronomy, Faculty of Agronomy, University of Forestry, 10 Kliment Ohridski Blvd, 1797 Sofia, Bulgaria; 10 Department of Plant Production, Faculty of Agriculture, Lebanese University, Beirut 1302, Lebanon

**Keywords:** abiotic stress, antioxidant enzymes, growth, *Lactuca sativa*, mineral ions, morphological traits, nitric oxide, photosynthetic pigments, physiological traits, stress mitigation

## Abstract

Salt stress negatively affects the growth, development, and yield of horticultural crops. Nitric oxide (NO) is considered a signaling molecule that plays a key role in the plant defense system under salt stress. This study investigated the impact of exogenous application of 0.2 mM of sodium nitroprusside (SNP, an NO donor) on the salt tolerance and physiological and morphological characteristics of lettuce (*Lactuca sativa* L.) under salt stress (25, 50, 75, and 100 mM). Salt stress caused a marked decrease in growth, yield, carotenoids and photosynthetic pigments in stressed plants as compared to control ones. Results showed that salt stress significantly affected the oxidative compounds (superoxide dismutase (SOD), peroxidase (POD), catalase (CAT) and ascorbate peroxidase (APX)) and non-oxidative compounds (ascorbic acid, total phenols, malondialdehyde (MDA), proline, and H_2_O_2_) in lettuce. Moreover, salt stress decreased nitrogen (N), phosphorous (P), and potassium ions (K^+^) while increasing Na ions (Na^+^) in the leaves of lettuce under salt stress. The exogenous application of NO increased ascorbic acid, total phenols, antioxidant enzymes (SOD, POD, CAT, and APX) and MDA content in the leaves of lettuce under salt stress. In addition, the exogenous application of NO decreased H_2_O_2_ content in plants under salt stress. Moreover, the exogenous application of NO increased leaf N in control, and leaf P and leaf and root K^+^ content in all treatments while decreasing leaf Na^+^ in salt-stressed lettuce plants. These results provide evidence that the exogenous application of NO on lettuce helps mitigate salt stress effects.

## 1. Introduction

Among abiotic stresses, salt stress is considered as the most dangerous stress that disturbs growth and yield, causes physiological disorders, and results in the death of plants [1]. Salinity is a more limiting factor increasing from arid to semiarid regions all over the globe [2]. Globally, approximately 2000 ha of arable land are moving towards production loss daily due to excessive salt concentrations [3]. These production losses were estimated to be around 10–25% [4]. The higher salt concentrations in irrigation water are the major cause of salt buildup in agricultural lands and plant root zones, inducing stunted growth [5]. Particularly, sodium and chloride concentrations increase within plant cell compartments growing in saline conditions [6]. Soil soluble salts and water sodium chloride are the main salinity-inducing factors in semiarid and hot regions [7,8]. High salt accumulations result from the salinity of irrigation water in plant cells as well as in the soil [9]. Salt stress increases oxidative injury in plants, which further damages the photosynthetic machinery and stomata regulation [10]. Under extreme NaCl stress conditions, the production of reactive oxygen species (ROS) is enhanced, which threatens normal plants. These ROS have the ability to rupture photosynthetic pigments [11]. The production of antioxidants, i.e., SOD, POD and CAT activities, non-enzymatic compounds, i.e., ascorbic acid, phenolic content, different sugars, MDA, H_2_O_2_, and osmolytes, i.e., proline, GB, and APX, were enhanced within plants under extreme saline conditions [11]. Different antioxidants have the capability to eliminate ROS and improve plant immunity under harsh environmental conditions [10,12]. Different strategies, i.e., balanced nutrient application, timely irrigation, and application of different chemicals can be used to reduce the negative effects of salt stress. The application of sodium nitroprusside is considered an important nutrient element to reduce the adverse effects of abiotic stresses. Under salt stress, sodium nitroprusside can be a suitable fertilizer for improvement of the salt tolerance mechanism in plants [13]. Foliar application could be considered the most effective method to activate the antioxidant system and regulate physiological processes in plants under stress [14].

Nitric oxide is an important signaling molecule that is involved in different plant processes, e.g., seed dormancy, seed emergence, stomata regulation, photosynthesis, growth of pollen tubes, flowering, and fruiting [12,15]. Sodium nitroprusside (SNP; an NO donor) modulated the damaging effects of ROS on plant growth by improving the antioxidant enzyme activities [16,17]. The application of nitric oxide was also recorded in numerous crops, e.g., tomato [18], cucumber [19], chickpea [20], soybean [21], spinach [22] and pak choi [17], being effective in improving the salt tolerance mechanism within plants [23]. Moreover, it was also found to be effective in the enhancement of tolerance mechanisms in higher plants growing under harsh climatic conditions [24].

Lettuce (*Lactuca sativa* L.) is an important crop rich in iron that is crucial for human healthy life; it is mainly used in salads in the daily diet [25]. However, its cultivation faces high salinity risks [26]. Lettuce faces very low production due to poor soil, harsh environmental conditions, and irrigation with poor-quality water. Its production can be increased via the enhancement of salt tolerance through the application of required nutrients. Soil compaction due to heavy fertilization is also a major cause of poor production.

Based on the aforementioned, foliar spray can be effective for sustainable crop production. The protective role of NO in plants was previously hypothesized to decrease the adverse effect of salt stress in lettuce without any real evidence. However, until now, little information has been available regarding the effect of exogenous application of NO on oxidative damage and antioxidant enzyme activities in lettuce under salt stress. Therefore, the present work aimed to (a) evaluate the growth and yield of lettuce plants under different levels of salinity with and without NO application, and (b) evaluate the effect of salt stress with and without NO application on relative water content (RWC), photosynthetic pigments, ascorbic acid, oxidative and non-oxidative compounds, and macro-elements of lettuce plants.

## 2. Results

### 2.1. Growth and Yield Traits

**Figure 1:**
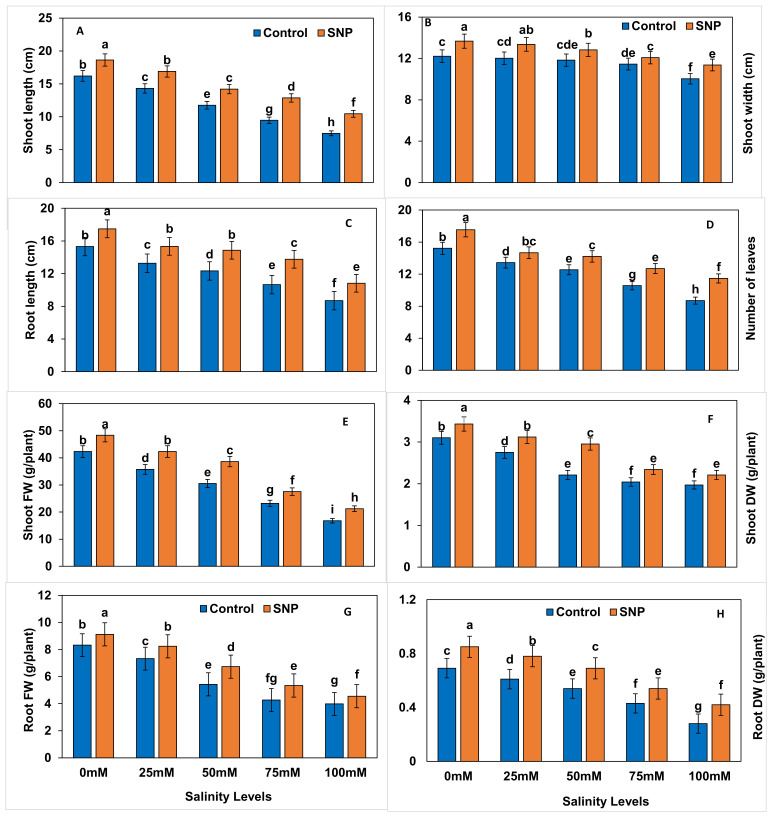
Effect of exogenous application of nitric oxide on the growth parameters, i.e., shoot length (**A**), shoot width (**B**), root length (**C**), number of leaves (**D**), shoot fresh weight (**E**), shoot dry weight (**F**), root fresh weight (**G**), and root dry weight (**H**) of lettuce under different levels of salt stress. The data presented are means ± SEs (n = 3). Different lower-case letters refer to statistically significant difference (*p* < 0.05) according to Duncan multiple range test (DMRT).

The effect of foliar application of nitric oxide on growth and yield traits of lettuce under different salinity levels (0 mM, 25 mM, 50 mM, 75 mM, and 100 mM) was investigated. Results showed that salt stress significantly (*p* < 0.01) affected plant growth and biomass, particularly when 100 mM salt were applied (Figure 1). SNP foliar application significantly (*p* < 0.01) increased shoot length (14.3–28.6%), shoot width (8.3–13.6%), root length (15.4–30.0%), number of leaves (11.5–35.3%), shoot fresh weight (FW) (20.0–27.3%), shoot dry weight (DW) (9.7–31.8%), root fresh weight (12.5–23.8%), and root dry weight (11.1–60.0%) of lettuce under 25-, 50-, 75-, and 100-mM salt stress (Table 1). Salinity treatments induced significant reductions (*p* < 0.01) in growth and yield traits of lettuce plants, by 9.7–68.7% (except for shoot width at 25- and 50-mM salinity levels) (Table 2). However, NO application reduced those percentages to a range of 3.7–56.2% when compared to control (Table 3). Table 4 lists the differences between salinity treatments with and without NO application (with–without) in terms of growth and yield traits. Difference values outlined significantly reduced percentages (*p* < 0.01) in terms of shoot length (−10.9 to −4.4%) with 25-, 75-, and 100-mM salinity treatments compared to control. The percentage difference in shoot width was significantly reduced (*p* < 0.01) by 2.4–5.2% with 75- and 100-mM salinity treatments compared to control. Similarly, the percentage difference in root length was significantly reduced (*p* < 0.01) by 2.4–7.1% with 50-, 75-, and 100-mM salinity treatments compared to control. The percentage difference in number of leaves was significantly increased (*p* < 0.01) by 3.3–4.3% with 25- and 50-mM salinity treatments and decreased (*p* < 0.01) by 5.7% with 100-mM salinity treatment compared to control. The percentage difference in shoot fresh weight was significantly reduced (*p* < 0.01) by 2.6–6.3% with 25-, 50-, and 100-mM salinity treatments compared to control, whereas the percentage difference in shoot dry weight was significantly reduced (*p* < 0.01) by 14.3% only with 50-mM salinity treatment compared to control. The percentage difference in root fresh weight was significantly reduced (*p* < 0.01) by 4.9–7.7% with 50- and 75-mM salinity treatments compared to control. The percentage difference in shoot width was significantly reduced (*p* < 0.01) by2.5–15.8 with all salinity treatments compared to control.

### 2.2. Relative Water Content

**Figure 2:**
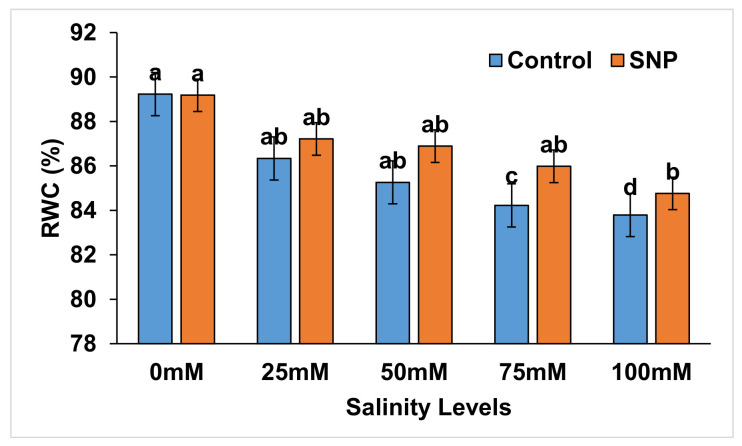
Effect of exogenous application of nitric oxide on the relative water content (%) in lettuce under different levels of salt stress. The data presented are means ± SEs (n = 3). Different lower-case letters refer to statistically significant difference (*p* < 0.05) according to Dunc multiple range test (DMRT).

Figure 2 shows that, without SNP foliar application, the relative water content (RWC) of lettuce was significantly decreased (*p* < 0.01) under salt stress by 2.5–5.0% compared to control. SNP foliar application showed no significant effect on the control, 25 mM, and 50 mM samples, whereas it increased RWC by 2.4 (*p* < 0.01) and 1.8% (*p* < 0.01) under the 75- and 100-mM salinity levels, respectively (Table 5). Salinity treatments of 75- and 100-mM yielded significant reductions (*p* < 0.01) in the relative water content of lettuce plants (by 6.0–6.5%) without NO application compared to control (Table 6), whereas NO application indicated a significant decrease (*p* < 0.01) in such effects, by 5.4%, with the 100-mM salt-stressed lettuce plants in comparison to control (Table 6). Table 7 lists the differences between salinity treatments with and without NO application (with–without) in terms of relative water content. Difference values indicated non-significant reduced percentages (*p* > 0.05) in terms of the analyzed parameter (−1.8 to −1.0%) with all salinity treatments compared to control.

### 2.3. Photosynthetic Pigments

**Figure 3:**
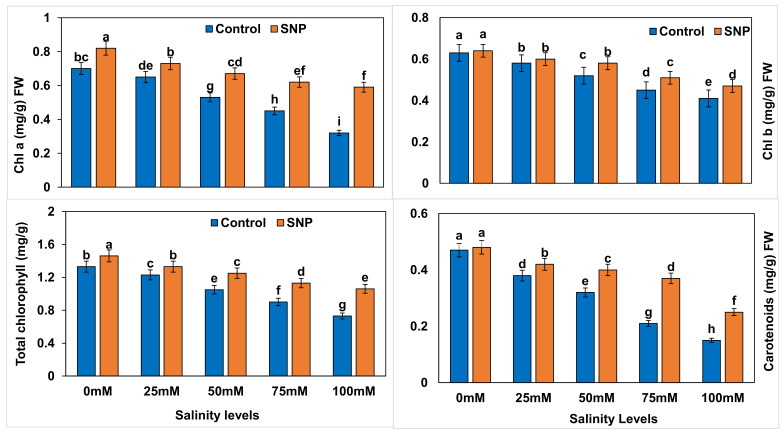
Effect of exogenous application of nitric oxide on the chlorophyll a, chlorophyll b, total chlorophyll and carotenoid content in lettuce under different levels of salt stress. The data presented are means ± SEs (n = 3). Different lower-case letters refer to statistically significant difference (*p* < 0.05) according to Duncan multiple range test (DMRT).

Photosynthetic pigments, i.e., chlorophyll a (*Chl a*), chlorophyll (*Chl b*), total chlorophyll, and carotenoid contents, in lettuce were significantly decreased (*p* < 0.01) with increased salinity levels (Figure 3). Chlorophyll a (*Chl a*) was significantly increased in SNP-treated lettuce by 15.4–93.9%, with the highest improvement noted with the 100 mM salinity level. Foliar application of SNP did not significantly affect *Chl b* in the control and 25 mM salinity-treated lettuce (*p* > 0.05), whereas it significantly enhanced (*p* < 0.01) *Chl b* by 11.1–15.7% in the 50, 75, and 100 mM-treated samples. Total chlorophyll (*Total Chl*) content significantly increased (*p* < 0.01) by 8.3–50.0% with foliar application of SNP to plants under salt stress. This application did not significantly affect the carotenoid (Ct) content in control plants (*p* > 0.05), whereas stressed plants showed a significant increase (*p* < 0.01) in carotenoid content by 7.7–80.0% with SNP foliar application (Table 8). Salinity treatments showed significant reductions (*p* < 0.01) in *Chl a*, *Chl b*, *Total Chl*, and Ct contents in lettuce plants by 6.9–69.6% without NO application compared to control (Table 9), whereas NO application significantly reduced (*p* < 0.01) those differences to 6.5–48.9% in comparison to control (Table 10). Table 11 lists the differences between salinity treatments with and without NO application (with–without) in terms of photosynthetic pigments. Difference values indicated significantly reduced percentages (*p* < 0.01) in terms of *Chl a* (–26.3 to –10.1%) with the 75- and 100-mM salinity treatments compared to control. The percentage differences in *Chl b* and Ct were significantly reduced (*p* < 0.01) by 6.5–11.4% and 6.8–35.2% with all salinity treatments, respectively, compared to control. Similarly, the percentage difference in *Total Chl* was significantly reduced (*p* < 0.01) by 11.3–19.4% with the 75- and 100-mM salinity treatments compared to control.

### 2.4. Ascorbic Acid Content

**Figure 4:**
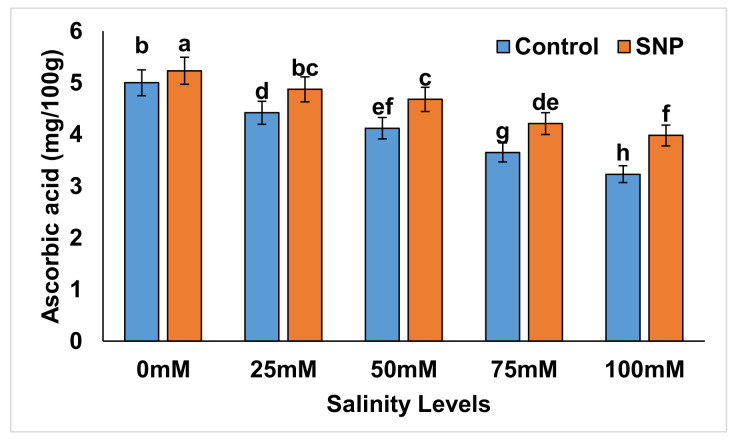
Effect of exogenous application of nitric oxide on the ascorbic acid content in lettuce under different levels of salt stress. The data presented are means ± SEs (n = 3). Different lower-case letters refer to statistically significant difference (*p* < 0.05) according to Duncan multiple range test (DMRT).

Without SNP foliar application, the ascorbic acid content was significantly decreased (*p* < 0.01) in salt-stressed lettuce plants by 10.0–40.0% compared to control (Figure 4). The foliar application of SNP significantly (*p* < 0.01) increased this content in stressed lettuce plants by 9.1–21.9% (Table 12). Salinity treatments caused significant reductions (*p* < 0.01) in the ascorbic acid content of lettuce plants by 12.0–36.0% without NO application compared to control (Table 13), whereas NO application significantly reduced (*p* < 0.01) such difference to 6.7–23.8% in salt-stressed lettuce plants in comparison to control. Table 14 lists the differences between salinity treatments with and without NO application (with–without) in terms of ascorbic acid content. Difference values indicated significantly reduced percentages (*p* < 0.01) in terms of this parameter (−12.2 to −5.3%) with all salinity treatments compared to control.

### 2.5. Oxidative Compounds

**Figure 5:**
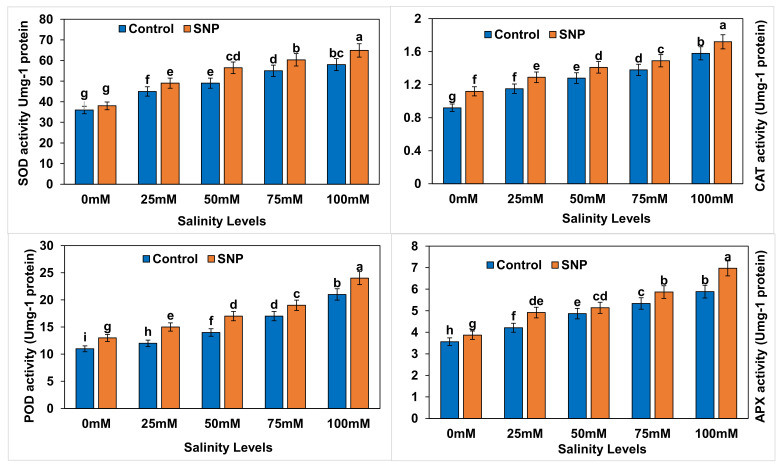
Effect of exogenous application of nitric oxide on the superoxide dismutase (SOD) enzyme activity, catalase (CAT) enzyme activity, peroxidase (POD) enzyme activity and ascorbate peroxidase (APX) enzyme activity in lettuce under different levels of salt stress. The data presented are means ± SEs (n = 3). Different lower-case letters refer to statistically significant difference (*p* < 0.05) according to Duncan multiple range test (DMRT).

Plant defense-related activities, i.e., SOD, POD, CAT and APX, were significantly increased (*p* < 0.01) in lettuce with increased salinity levels compared to control (Figure 5). Although foliar application of SNP did not significantly affect (*p* > 0.05) the SOD activity in control plants, it significantly enhanced (*p* < 0.01) this activity in lettuce plants under salt stress by 7.1–16.3%. CAT, POD, and APX activities significantly increased (*p* < 0.01) with foliar application of SNP to stressed lettuce plants by 8.7–15.4%, 11.8–25.0%, and 6.3–19.0%, respectively (Table 15). Salinity treatments caused significant increases (*p* < 0.01) in oxidative compounds in lettuce plants by 9.1–90.9% without NO application compared to control (Table 16). Additionally, such application significantly increased (*p* < 0.01) those differences to 15.4–94.6% (except for CAT) in comparison to control (Table 17). Table 18 lists the differences between salinity treatments with and without NO application (with–without) in terms of oxidative compounds. Difference values indicated significantly increased percentages (*p* < 0.01) in terms of SOD (3.9–13.9%) with all salinity treatments compared to control. The percentage difference in CAT was significantly reduced (*p* < 0.01) by 9.6–18.2% with all salinity treatments compared to control, whereas the percentage difference in root length was significantly increased (*p* < 0.01) by 3.5–6.3% with the 25-, 50-, and 100-mM salinity treatments compared to control. Similarly, the percentage difference in APX was significantly increased (*p* < 0.01) by 12.2–18.9% with the 25-, 75-, and 100-mM salinity treatments.

### 2.6. Non-Oxidative Compounds

**Figure 6:**
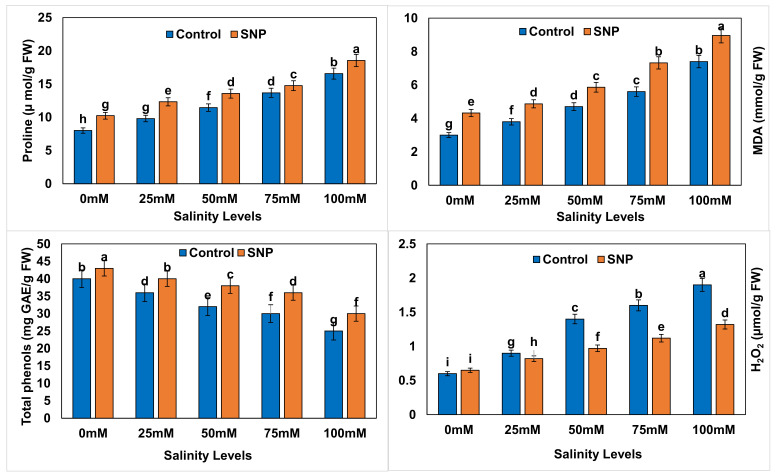
Effect of exogenous application of nitric oxide on the proline content, malondialdehyde content, total phenols, and H_2_O_2_ content in lettuce under different levels of salt stress. The data presented are means ± SEs (n = 3). Different lower-case letters refer to statistically significant difference (*p* < 0.05) according to Duncan multiple range test (DMRT).

The contents of non-oxidative compounds, i.e., proline, malondialdehyde (MDA), and H_2_O_2_, were significantly increased (*p* < 0.01) in lettuce with increased salinity levels, whereas total phenols content significantly decreased (*p* < 0.01) under the same conditions (Figure 6). Proline and MDA contents significantly increased (*p* < 0.01) by 7.7–27.8% and 20.5–28.9%, respectively, with SNP foliar application to stressed lettuce plants. The foliar application of SNP resulted in a significant increase (*p* < 0.01) in total phenols content by 14.3–24.1% in lettuce plants under salt stress. Although foliar application of SNP did not significantly affect (*p* > 0.05) the H_2_O_2_ content in control plants, it significantly decreased (*p* < 0.01) this content by 12.5–31.2% in salt-stressed lettuce plants (Table 19). Salinity treatments caused significant increases (*p* < 0.01) in non-oxidative compounds in lettuce plants (9.1–90.9%) without NO application compared to control (except for TP) (Table 20). Additionally, NO application significantly increased (*p* < 0.01) those differences to 11.9–114.3% (except for TP) in comparison to control (Table 21). Table 22 lists the differences between salinity treatments with and without NO application (with–without) in terms of non-oxidative compounds. Difference values indicated significantly reduced percentages (*p* < 0.01) in terms of proline (−28.6 to −10.7%) with all salinity treatments compared to control. The percentage difference in MDA was significantly reduced (*p* < 0.01) by 20.2–39.3% with 25-, 50-, and 100-mM salinity treatments compared to control. Similarly, the percentage of differences in total phenols and H_2_O_2_ were significantly reduced (*p* < 0.01) by 4.1–9.6% and 29.5–128.1% with all salinity treatments compared to control.

### 2.7. Macronutrient Elements

**Figure 7:**
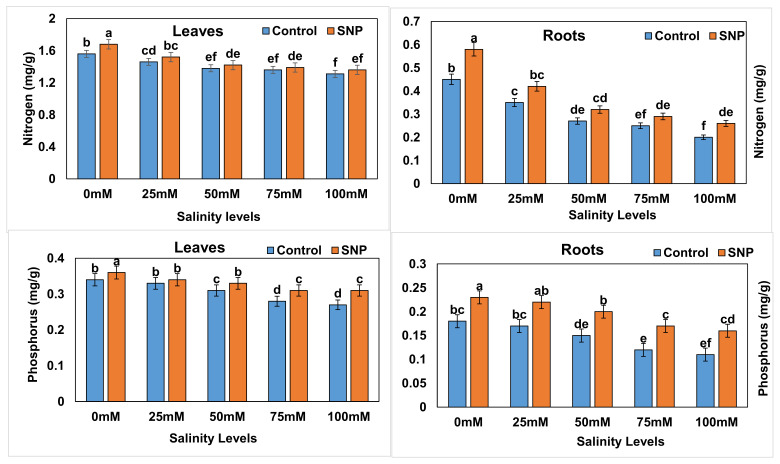
Effect of exogenous application of nitric oxide on leaf and root nitrogen and phosphorous contents in lettuce under different levels of salt stress. The data presented are means ± SEs (n = 3). Different lower-case letters refer to statistically significant difference (*p* < 0.05) according to Duncan multiple range test (DMRT).

**Figure 8:**
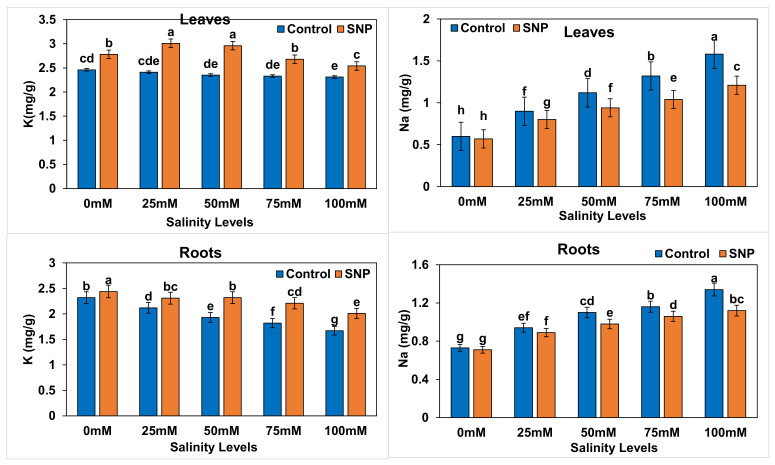
Effect of exogenous application of nitric oxide on sodium (Na^+^) and potassium (K^+^) contents in the leaves and roots of lettuce under different levels of salt stress. The data presented are means ± SEs (n = 3). Different lower-case letters refer to statistically significant difference (*p* < 0.05) according to Duncan multiple range test (DMRT).

Leaf nitrogen and leaf phosphorus contents were significantly decreased (*p* < 0.01) in lettuce plants under salt stress compared to the control (Figure 7). The foliar application of SNP increased (*p* < 0.01) leaf nitrogen content by 9.7% in control and by 0.34–0.40% (*p* > 0.05) in salt-stressed lettuce plants. Although this application did not significantly affect (*p* > 0.05) the leaf phosphorus content in 25 mM salt-stressed plants, it significantly increased (*p* < 0.01) this content by 5.7–15.4% in the control and 50-, 75-, and 100 mM salt-stressed lettuce plants (Table 23), whereas root nitrogen and root phosphorus contents were significantly decreased (*p* < 0.01) in lettuce plants under salt stress (except for 25 mM; *p* > 0.05) compared to the control (Figure 8). The foliar application of SNP increased (*p* < 0.01) root nitrogen content by 26.7 and 25.0% in control and 100 mM salt-stressed lettuce plants, respectively, and by 17.6–28.0% (*p* > 0.05) in 25-, 50-, and 75 mM salt-stressed lettuce plants. Although this application did not significantly affect (*p* > 0.05) the root phosphorus content in 25 mM salt-stressed plants, it significantly increased (*p* < 0.01) this content by 27.8–50.0% in the control and the 50-, 75-, and 100 mM salt-stressed lettuce plants (Table 23). Compared to control, salinity treatments applied to lettuce plants showed significant decreases (*p* < 0.01) in leaf and root nitrogen (8.2–55.5%) and leaf and root phosphorus contents (14.3–44.4%) (except for 25-mM) without NO application (Table 24). The latter significantly accentuated (*p* < 0.01) those decreases to 11.8–56.9% for leaf and root nitrogen and leaf phosphorus contents and to 13.0–34.8% for root phosphorus content (except for 25-mM) in comparison to control (Table 25). Table 26 lists the differences between salinity treatments with and without NO application (with–without) in terms of leaf and root N and *p*. Difference values indicated significantly increased and decreased percentages (*p* < 0.01) in terms of leaf N and root P (3.0 to 4.6% and 2.4 to 9.6%, respectively) with all salinity treatments compared to control. The percentage difference in root N was significantly increased (*p* < 0.01) by 6.5% with only the 50-mM salinity treatment compared to control, whereas the percentage of difference in leaf P was significantly reduced (*p* < 0.01) by 4.0–6.8% with the 75- and 100-mM salinity treatments compared to control.

Potassium ions (K^+^) in lettuce roots decreased significantly (*p* < 0.01) when plants were under salt stress, whereas K^+^ in lettuce leaves decreased significantly (*p* < 0.01) only in 100 mM salt-stressed plants. Sodium ions (Na^+^) in lettuce leaves and roots were significantly increased (*p* < 0.01) in lettuce plants under salt stress compared to the control (Figure 8). Leaf K^+^ did not show significant variation between most treatments except with the 100 mM salinity level (*p* < 0.01). The foliar application of SNP significantly increased K^+^ content in both leaves and roots of lettuce under salt stress by 6.7–26.1% and 9.5–22.2%, respectively. It is noteworthy that leaf K^+^ of 25 mM and 50 mM salt-stressed lettuce plants without SNP application was higher than in the control by 11.1 and 7.4%, respectively. Although the foliar application of SNP did not significantly affect (*p* > 0.05) leaf and root Na^+^ contents in control plants, it significantly reduced (*p* < 0.01) them by 11.8–22.6% and 5.3–18.5%, respectively in lettuce plants under salt stress (Table 27). Plants treated at the 100 mM-salinity level showed a significant decrease (*p* < 0.01) by 7.1% in leaf K^+^ content compared to control when NO was not applied (Table 28), whereas with NO application, leaf K^+^ first significantly increased (*p* < 0.01) by 5.5–9.1% with the 25- and 50-mM salinity levels, did not significantly (*p* > 0.05) vary with the 75-mM salinity level, and significantly decreased (*p* < 0.01) by 9.1% with the 100-mM salinity level compared to control (Table 29). Leaf Na^+^ significantly increased (*p* < 0.01) in salt-stressed lettuce plants compared to control by 41.7–166.7% without NO application and by 36.4–118.2% with NO application. In a similar trend, root K^+^ significantly decreased (*p* < 0.01) in salt-stressed lettuce plants compared to control by 8.7–30.4% without NO application and by 2.1–20.8% when NO was applied. Root Na^+^ significantly increased (*p* < 0.01) in salt-stressed lettuce plants compared to control by 22.0–73.3% without NO application and by 21.4–57.1% when NO was applied. Table 30 lists the differences between salinity treatments with and without NO application (with–without) in terms of leaf and root Na^+^ and K^+^. Difference values indicated significantly increased percentages (*p* < 0.01) in terms of leaf K^+^ (2.0 to 3.2%) with 25- and 100-mM salinity treatments compared to control. The percentage differences in leaf Na^+^ and root K^+^ were significantly reduced (*p* < 0.01) by 5.3–48.5% and 4.5–15.6%, respectively, with all salinity treatments compared to control. Similarly, the percentage difference in root Na^+^ was significantly reduced (*p* < 0.01) by 3.3–16.2% with the 50-, 75-, and 100-mM salinity treatments compared to control.

## 3. Discussion

Salinity-induced osmotic and ionic stresses cause oxidative damage and produce ROS, impairing photosynthesis and cellular membrane damage, and an overall reduction in plant biomass production [14]. Salt stress affects the growth and development of plants and ultimately reduces their yield [27,28,29]. The role of NO as a signaling molecule in salt stress responses has been well documented [30,31]. This study investigated the effect of exogenously applied NO on the growth, enzymatic activities, and physiology of lettuce under salt stress conditions.

Lettuce is considered sensitive to salinity, which reduces its growth, root and shoot length, yield and total biomass [32]. In this study, under salt stress, the plant growth and yield of lettuce were markedly reduced. Both shoot and root length were significantly affected by the salinity exposure of lettuce plants. Similar reductions in the growth parameters were observed earlier in lettuce due to high salt concentrations in water and soil [1,33,34,35]. The higher leaf uptake of salts inhibits plant growth by reducing the nutrient supply to the plants [36]. Our results showed that NO exogenous application to lettuce plants enhanced plant fresh weight, number of leaves, shoot dry weight and yield, and ultimately enhanced tolerance in lettuce under salt stress. Similar improvements in growth parameters such as root length and shoot fresh and dry weight were observed in basil [37], pak choi [17], and wheat [30] as well as in mung bean under salt stress [38] with NO exogenous application. NO, as a signaling molecule, plays a crucial role in the improvement of crop growth under stress and normal conditions [37]. It is worth noting that the increases in growth traits of control (0 mM) plants were more pronounced when NO was applied. This outlines the positive impact of combining full Hoagland solution with SNP treatment, suggesting a cumulative availability of N concentrations for lettuce growth and development. The comparison between the “with NO” and “without NO” applications and the evaluated differences between them revealed that NO application significantly minimized the reduction in SL (25, 75, and 100 mM), SW (75 and 100 mM), RL (50, 75, and 100 mM), NL (100 mM), SFW (25, 50, and 100 mM), SDW (50 mM), RFW (50 and 75 mM), and RDW (all salinity levels) of salt-stressed lettuce plants compared to the control. This outlines the positive impact of NO application on the growth and yield traits of lettuce plants under salt stress; it improved the tolerance of lettuce to salinity by activating the stress tolerance mechanisms of the crop. 

Salt stress negatively affected the absorption of water due to the accumulation of salts in the root zone, which decreases water potential and reduces the availability of water to plants. This decrease was more accentuated with increased salinity levels. A similar result was previously reported by Ahmed et al. [1], who described the decrease in RWC of lettuce shoot under salt stress. The negative effect of salts on the relative water content of shoots has also been reported in many other studies [35,39,40,41]. On the other hand, our findings showed that NO application increased leaf RWC content in salt-stressed lettuce plants. Such increase was previously reported on tomato leaves [42], rapeseed leaves [37], and pepper leaves [43] with NO exogenous application. However, the comparison between “with NO” and “without NO” applications and the evaluated differences between them revealed that NO application did not show any significant reduction in RWC of salt-stressed lettuce in comparison with control. This can be explained by the ability of NO to facilitate better management of water content by the plant, thus reducing its loss via transpiration, evapotranspiration, etc.

Our findings revealed a decrease in chlorophyll a, b, total chlorophyll and carotenoid contents in lettuce leaves with increased salinity levels. This decrease was previously reported in lettuce under salt stress [44,45,46]. Higher salt accumulation in the leaves might have oxidized the chlorophyll and chloroplast, resulting in reduced concentrations of pigment protein [47]. Enhanced ROS production under salt stress might have reduced the photosynthetic activity and increased the degradation of these pigments [48]. Chlorophyll molecules are essential for photosynthesis; any reduction in leaf chlorophyll content might lead to a perturbation in the photosynthesis mechanism and ultimately a decrease in yield [46]. Herein, the exogenous application of NO enhanced these photosynthetic pigments in both normal and salt-stressed lettuce leaves, and carotenoids in salt-stressed ones. Similar findings were observed in broccoli [49], mung bean [37], rapeseed [38], tomato [18], and wheat [29]. The comparison between “with NO” and “without NO” applications and the evaluated differences between them revealed that NO application significantly minimized the reduction in *Chl a* and *Total Chl* (75 and 100 mM), *Chl b* and Ct (in all salinity treatments) of salt-stressed lettuce plants compared to control. This can be explained by the fact that NO improves the stomatal conductivity, thus leading to better photosynthetic efficiency and increased carotenoid content.

Our study showed that salt stress significantly increased the activities of SOD, POD, CAT and APX antioxidant enzymes in lettuce as compared to the control. These antioxidant enzymes protect the cell membrane from oxidative damage and provide salt tolerance by scavenging ROS [49]. ROS can provoke severe oxidation, which can be controlled with the production of enzymatic and non-enzymatic antioxidants in plants under stress [1]. Previous studies reported the role of these antioxidant enzymes in scavenging ROS under salt stress in lettuce [50,51,52]. ROS can cause lipid peroxidation and oxidative damage to lipids and nucleic acid in lettuce [51]. A further increase in these antioxidant enzymes in lettuce under salt stress was recorded in this study with foliar application of NO. NO might have reduced the accumulation of ROS and protected cells from oxidative damage [43,49]. The increase in enzymatic activity responsible for ROS scavenging with NO application has been observed in various crops such as chickpea [20], cucumber [19], maize [31], pak choi [17], and tomato [23] under salt stress. The comparison between “with NO” and “without NO” application and the evaluated differences between them revealed that NO application significantly minimized the reduction in CAT of all salt-stressed plants in comparison with control. This finding also underscores the improved ROS scavenging as mentioned earlier.

Non-enzymatic antioxidants such as ascorbic acid and total phenols are also effective in reducing cell membrane damage from oxidative stress due to their antioxidative potential in plants [52]. In this study, the ascorbic acid content in lettuce decreased with the increase in salinity levels, whereas NO foliar application on lettuce plants enhanced ascorbic acid content. Such increases were observed earlier with the exogenous application of NO on broccoli [49], pea [53], and rice [54] under salt stress. Moreover, non-oxidative compounds, i.e., proline and the MDA content, in the leaves increased in this study with the increase in salinity levels as compared to control plants. However, the exogenous application of NO showed a further decrease in H_2_O_2_ in the leaves of lettuce under salt stress. Earlier studies reported an increase in proline content associated with increased salinity [12,17,41,49,55]. Under salt stress, proline protects enzymes, photosynthetic machinery and cell membranes against oxidative stress, consequently increasing tolerance to salinity in plants [41]. Proline is a low molecular osmolyte that accumulates in plants, scavenges hydroxyl radicals, and protects protein and DNA against ROS [41]. Previous studies reported that the exogenous application of NO enhanced the proline content under salt stress in various crops such as broccoli [49], pepper [43], and rice [54]. Furthermore, NO decreases the accumulation of H_2_O_2_ and MDA in plants under salt stress [29,49]. The comparison between “with NO” and “without NO” application and the evaluated differences between them revealed that NO application significantly minimized the reduction in ascorbic acid, proline, total phenols, H_2_O_2_, and MDA contents of salt-stressed lettuce plants compared to the control. This can be explained by the fact that NO helps in the synthesis of ascorbic acid, acting as a signaling molecule. Additionally, such findings can be explained by the close inter-relationship between NO and proline synthesis during the full growing cycle of lettuce plants under salt stress. Further, it is known that total phenols and MDA contents vary in parallel; this shows how NO acted efficiently by increasing them and reducing the differences between salinity treatments and control. Moreover, NO may have activated the H_2_O_2_ circulation in lettuce plants under salt stress, which was contrasted with reduced difference in such signaling molecule amount in comparison with the control.

Salt stress generally decreased leaf nitrogen and phosphorus of lettuce as compared to the control, whereas NO application only increased leaf phosphorus content (*p* < 0.01). Moreover, in general, salt stress significantly decreased (*p* < 0.01) root nitrogen and root phosphorus, except in the 25 mM saline-treated plants (*p* > 0.05). However, NO application increased (*p* < 0.01) root nitrogen in the control and 100 mM saline-treated plants and root phosphorus in all treatments except the 25 mM saline-treated plants (*p* > 0.05). This could be attributed to the role played by NO during photosynthesis, cell membrane protection against ROS, and thus, the uptake of these elements in higher amounts by leaves and roots. Furthermore, salt stress increased the concentrations of Na^+^ and reduced the K^+^ content in lettuce plants. The increase in Na^+^ was more pronounced with the increase in salinity as compared to control plants. A similar trend was found in an earlier study on lettuce by Zhang et al. [41], who also found an increase in Na^+^ and a decrease in K^+^ contents under salt stress. The excessive uptake of Na^+^ by plants can cause a devastating effect on the cell membrane by lowering the amount of K^+^, thus leading to an imbalance in cell homeostasis [41,49,54]. K^+^ deficiency in plants caused by a higher Na^+^ uptake can lead to reduced growth and production in response to lower photosynthesis, stomatal closure and decreased enzymatic activities [41]. In the current study, the exogenous application of NO enhanced the uptake of K^+^ and decreased Na^+^ uptake under salt stress conditions. Maintaining high K^+^ and low Na^+^ might facilitate the nutritional balance and protect from ion toxicity [56]. Our results are in agreement with those of previous studies in which NO application increased K^+^ and decreased Na^+^ in various crops such as broccoli [49] and wheat [57] under salt stress. Our findings revealed that NO maintains the nutritional balance, which might have improved the overall growth of lettuce under salt stress. The comparison between “with NO” and “without NO” applications and the evaluated differences between them revealed that NO application significantly minimized the reduction in leaf P (75 and 100 mM) and root P (all treatments) of salt-stressed lettuce plants compared to control. Similarly, the reduction in leaf K^+^ (75 mM), root Na^+^ (50, 75, and 100 mM), and leaf Na^+^ and root K^+^ (all salinity treatments) was minimized with NO application in salt-stressed lettuce plants compared to control. This can be explained by the increase in nutrient uptake and ion exchange in lettuce as a result of NO application.

## 4. Materials and Methods

### 4.1. Experimental Site and Materials

The current study was conducted at the Vegetable Research Area of the Department of Horticulture, Bahauddin Zakariya University, Multan, Pakistan between October 2019 and March 2020. The pot experiment was performed in a moveable warehouse covered with polythene sheet to protect the plants from heavy rain. Sown lettuce cv. Grand Rapid seeds were purchased from More Green Company, Lahore, Pakistan. Two thousand lettuce seeds from the selected cultivar were sown in 100 plastic pots (20 cm diameter and 30 cm depth). Each pot was filled with 2 kg of thoroughly washed river sand. Growing pots had drainage holes covered with a piece of muslin cloth at the bottom. Soil leaching was performed prior to the experiment to flush through all previously present salt (NaCl) in the sand. Each pot was irrigated with 10 mL full-strength Hoagland’s nutrient solution (pH 6.5) on alternate days, based on a pilot study performed before launching the present experiment. Lettuce seedlings were allowed to emerge for 1 week and were then thinned to 6 plants per pot. One week after, the plants were thinned to 3 per pot, chosen based on a uniform size, and placed equidistantly. Salt was artificially applied to the pots at various concentrations (0, 25, 50, 75 and 100 mM). After 14 days of seeding, the respective salinity levels were applied to the corresponding pots on subsequent days on an alternate interval to avoid osmotic shock. All lettuce seeds/plants were irrigated with 100 mL distilled water on alternate days, which avoided an increase in salt concentrations. Such irrigation criteria were followed on the basis of the aforementioned pilot study.

### 4.2. Experiment Design

The experiment was conducted following a completely randomized design (CRD) to reduce any effect of location or exposure to sunlight or wind. A two-way factorial design of salt and SNP was adopted, with three replications per treatment (3 pots per treatment; 20 seeds per pot). The control plants were mock sprayed with only Tween 20 (surfactant) dissolved in distilled water. Spraying of SNP (0.2 mM) was performed twice at days 15 and 30 of seedling establishment on lettuce leaves. SNP (100 mL/pot) prepared on a Tween 20 aliquot (as a wetting agent) was applied to lettuce leaves only, using a handled manual atomizer. All plants were harvested after 45 days, and morphological and biochemical attributes were recorded.

### 4.3. Laboratory Analysis

#### 4.3.1. Morphological and Yield-Related Attributes

**Figure 9:**
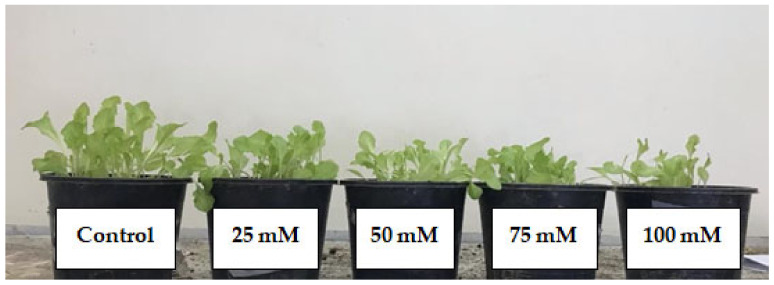
Effect of applied salinity levels on lettuce seedling growth and development.

Three plants, harvested from different pots, were randomly selected from each treatment for evaluation of morphological and yield-related attributes. Figure 9 shows the effect of salinity levels on growth and development of lettuce seedlings.

Shoot length (SL) and shoot width (SW) were measured using a measuring scale. Root length (RL) was measured with a Vernier caliper. The number of leaves (NL) was counted manually. Root (RFW) and shoot fresh (SFW) weights were determined using a digital weighing balance. Shoot (SDW) and root dry (RDW) weights were determined after drying in an oven at 70 °C for 48 h (to obtain a stable weight). *Relative water content* (RWC) in the leaves was determined using the formula (Equation (1)) of Karimi et al. [58].

(1) $$Relative\;water\;content\;\left(\%\right)=\frac{Fresh\;weight-Dry\;weight}{Turgid\;weight-Dry\;weight}\times 100$$

#### 4.3.2. Determination of Photosynthetic Pigments

Chlorophyll a (*Chl a*), chlorophyll b (*Chl b*), total chlorophyll (*Total Chl*), and *Carotenoid* contents were determined by grinding 0.5 g of fresh plant leaves in 10 mL acetone (80%) according to the method of Arnon [59] (Equations (2)–(5)). The prepared supernatant was kept at 4 °C overnight until analysis. The reading was recorded at 645 nm for *Chl a* and 663 nm for *Chl b* using a spectrophotometer. The *Carotenoid* (Ct) content of the extract was determined by reading the absorbance at 470 nm.

(2) Chl a mgg=100×A663×0.0127−A645×0.00269

(3) Chl b mgg=100×A645×0.0229−A663×0.00468

(4) Total Chl mgg=100×A645×0.0202+A663×0.00802

(5) Carotenoid mgg=1000×A470)−(3.27×Chl a−104×Chl b227

#### 4.3.3. Evaluation of Antioxidant Enzyme Activities

The activities of antioxidant enzymes including superoxide dismutase (SOD: EC 1.15.1.1), catalase (CAT: EC 1.11.1.6), peroxidase (POD: EC 1.11.1.7), and ascorbate peroxidase (APX: EC 1.11.1.11) were measured in lettuce leaves. Briefly, enzymatic activities were assessed by grinding leaf samples (1 g) in sodium phosphate buffer (50 mM: 1 mL: 7 pH) including 0.5 mmol EDTA. Samples were then centrifuged at 12,000 rpm for the separation of supernatant. After centrifugation at 12,000 rpm for 10 min at 4 ℃, the supernatant was removed, and readings for SOD, POD and CAT were noted using a spectrophotometer. Enzyme activity was measured following the method of Chen and Pan [60] and expressed as U g^−1^ FW, whereas ascorbate peroxidase (APX) activity was measured according to the method of Nakano and Asada [61].

#### 4.3.4. Measurement of MDA, Proline and H_2_O_2_ Contents

To determine the lipid peroxidation under salt stress, 1 g of leaf (FW) was homogenized in 3 mL TCA (0.1%) as described by Cakmak and Horst [62]. The malondialdehyde (MDA) content was calculated by subtracting the absorbance at 600 and 532 nm [62]. For proline (Pro) content determination, the youngest fresh leaf of lettuce (0.5 g) was extracted in a 5 mL sulfosalicylic acid (3%) following the method of Bates et al. [63], and reading was recorded at 520 nm using a UV/visible spectrophotometer. For the determination of hydrogen peroxide (H_2_O_2_) content, a fresh leaf (0.5 g) sample of lettuce was ground in 3 mL of 0.1% trichloroacetic acid (TCA). Then, reading was recorded at 390 nm using a spectrophotometer following the protocol of Velikova et al. [64].

#### 4.3.5. Measurement of Total Phenols

The total phenols (TP) content in lettuce leaves was determined as described by Martinez et al. [65]. Briefly, a 0.2 g leaf sample was extracted in 25 mL acetone (80%) solution and centrifuged at 10,000× *g* for 20 min. The extract (1 mL) was placed into a volumetric flask and mixed with 1 mL of Folin–Ciocalteu reagent and 5 mL of sodium bicarbonate. Then, the volume was made to 10 mL by adding distilled water. After vigorous vortex mixing, the flask was placed in the dark for 40 min to rest. TP content (mg GAE/g FW) was recorded at 730 nm using a spectrophotometer.

#### 4.3.6. Measurement of Ascorbic Acid 

The ascorbic acid (AA) concentration was measured as previously described by Ali et al. [66]. Briefly, a fresh leaf sample (2.5 g) was crushed in 100 mL oxalic acid (0.4% concentrated) and filtered. The supernatant was vortexed, then centrifuged at 6300× *g* for 10 min. Afterwards, the filtrate was titrated against standard dye (2,6-dichlorophenolindophenol) to a pink point. The AC concentration was expressed as mg/100 g on a fresh weight basis.

#### 4.3.7. Measurement of Macro-Elements 

Collected leaves and roots were washed and oven-dried (70 °C, 48 h); then, dried samples were ashed (500 °C) and digested (2 N HCl) in a digestion flask on a hot plate. The temperature was increased gradually from 50 °C to 200 °C, and the sample was heated until the material became colorless. Leaf and root nitrogen (N) contents were determined through Kjeldahl digestion of the dried sample [67]. Leaf and root phosphorus (P) contents were determined using the vanadomolybdate phosphoric acid method [68]. Moreover, leaf and root potassium (K) and sodium (Na) contents in control and salt-stressed lettuce plants were determined following the method of Enders and Lehmann [69]. Na^+^ and K^+^ concentrations were determined by a flame photometer (Jenway-PFP7, ELE Instrument Co., Ltd., Loveland, CO, USA).

### 4.4. Statistical Analysis 

Data were analyzed using Statistics 8.1 software. All means within each variable were statistically compared using the Duncan multiple range test (DMRT) at 5% probability level. Analysis of variance (ANOVA) test was performed to compare between treatments (different lower-case letters refer to statistically significant difference).

## 5. Conclusions

In the present study, the effect of nitric oxide foliar application on the shoot fresh and dry weight, root length, root fresh weight and dry weight, chlorophyll, carotenoids, macro-elements, antioxidant enzymes, and mineral ions of lettuce under salt stress conditions was investigated. Salinity drastically reduced the growth, leaf relative water content and yield of lettuce. However, NO exogenous application increased lettuce yield under salt stress conditions. Lettuce plants subjected to salt stress had lower antioxidant activity compared to those treated with NO. SNP foliar application also improved the uptake of phosphorus and potassium ions while reducing the uptake of sodium ions under salt stress. In addition, the activity of oxidative and non-oxidative compounds was improved in response to the exogenous application of NO on lettuce plants under salt stress, which mitigated the negative effect of salinity. The results suggest that the exogenous application of nitric oxide significantly contributed to the salt tolerance of lettuce plants, yielding significantly reduced differences in comparison with control in terms of yield and growth traits, photosynthetic pigments, ascorbic acid, CAT, non-oxidative compounds, and macronutrient elements. Further studies should investigate the potential role of NO in the mitigation of salt stress in other horticultural crops.

## Figures and Tables

**Table 1:** Increase in growth and yield traits with NO application.

Treatment	Percentage (%) Increase with NO Application							
	SL	SW	RL	NL	SFW	SDW	RFW	RDW
Control	33.3 *	8.3 *	13.3 *	16.7 *	14.3 *	9.7 *	15.8 *	21.4 *
25 mM	14.3 *	13.0 *	15.4 *	11.5 *	20.0 *	14.8 *	17.3 *	25.0 *
50 mM	18.2 *	13.6 *	20.0 *	12.0 *	22.6 *	31.8 *	18.2 *	40.0 *
75 mM	33.3 *	9.1 *	30.0 *	18.2 *	27.3 *	15.0 *	23.8 *	11.1 *
100 mM	28.6 *	10.0 *	23.5 *	35.3 *	23.5 *	15.8 *	12.5 *	60.0

SL: shoot length; SW: shoot width; RL: root length; NL: number of leaves; SFW: shoot fresh weight; SDW: shoot dry weight; RFW: root fresh weight; RDW: root dry weight; *: significant at *p* < 0.01.

**Table 2:** Decrease in growth and yield traits in salinity treatments without NO application compared to control.

Treatment	Percentage (%) Decrease without NO Application Compared to Control							
	SL	SW	RL	NL	SFW	SDW	RFW	RDW
25 mM	12.5 *	4.0 ns	13.3 *	10.0 *	15.3 *	9.7 *	9.8 *	12.5 *
50 mM	25.0 *	8.0 ns	16.7 *	16.7 *	27.1 *	29.0 *	29.3 *	25.0 *
75 mM	40.6 *	12.0 *	30.0 *	30.0 *	43.5 *	33.9 *	48.8 *	43.7 *
100 mM	53.1 *	20.0 *	43.3 *	40.0 *	58.8 *	37.1 *	52.4 *	68.7 *

SL: shoot length; SW: shoot width; RL: root length; NL: number of leaves; SFW: shot fresh weight; SDW: shoot dry weight; RFW: root fresh weight; RDW: root dry weight; ns: not significant; *: significant at *p* < 0.01.

**Table 3:** Decrease in growth and yield traits in salinity treatments with NO application compared to control.

Treatment	Percentage (%) Decrease with NO Application Compared to Control							
	SL	SW	RL	NL	SFW	SDW	RFW	RDW
25 mM	8.1 *	3.7 ns	11.4 *	14.3 *	12.5 *	8.8 *	8.9 *	8.2 *
50 mM	24.3 *	7.4 *	14.3 *	20.0 *	20.8 *	14.7 *	24.4 *	17.6 *
75 mM	29.7 *	9.6 *	22.9 *	28.6 *	43.7 *	33.8 *	41.1 *	41.2 *
100 mM	43.2 *	14.8 *	37.1 *	34.3 *	56.2 *	36.8 *	51.1 *	52.9 *

SL: shoot length; SW: shoot width; RL: root length; NL: number of leaves; SFW: shoot fresh weight; SDW: shoot dry weight; RFW: root fresh weight; RDW: root dry weight; *: significant at *p* < 0.01.

**Table 4:** Difference in terms of growth and yield traits in salinity treatments with/without NO application (with–without) compared to control.

Treatment	Percentage (%) Difference with/without NO Application (with–without) Compared to Control							
	SL	SW	RL	NL	SFW	SDW	RFW	RDW
25 mM	−4.4 *	−0.3 ns	−1.9 ns	+4.3 *	−2.8 *	−0.9 ns	−0.9 ns	−4.3 *
50 mM	−0.7 ns	−0.6 ns	−2.4 *	+3.3 *	−6.3 *	−14.3 *	−4.9 *	−7.4 *
75 mM	−10.9 *	−2.4 *	−7.1 *	−1.4 ns	+0.2 ns	−0.1 ns	−7.7 *	−2.5 *
100 mM	−9.9 *	−5.2 *	−6.2 *	−5.7 *	−2.6 *	−0.3 ns	−1.3 ns	−15.8 *

SL: shoot length; SW: shoot width; RL: root length; NL: number of leaves; SFW: shoot fresh weight; SDW: shoot dry weight; RFW: root fresh weight; RDW: root dry weight; +: increase; −: decrease; ns: not significant; *: significant at *p* < 0.01.

**Table 5:** Increase in relative water content with NO application.

Treatment	Percentage (%) Difference with/without NO Application
	RWC
Control	0 ns
25 mM	0.6 ns
50 mM	1.2 ns
75 mM	2.4 *
100 mM	1.8 *

RWC: relative water content; ns: not significant; *: significant at *p* < 0.01.

**Table 6:** Decrease in relative water content in salinity treatments with NO application compared to control.

Treatment	Percentage (%) Decrease with/without NO Application Compared to Control	
	RWC (without NO Application)	RWC (with NO Application)
25 mM	3.5 ns	2.5 ns
50 mM	4.7 ns	2.9 ns
75 mM	6.0 *	4.2 ns
100 mM	6.5 *	5.4 *

RWC: relative water content; ns: not significant; *: significant at *p* < 0.01.

**Table 7:** Difference in relative water content with/without NO application (with–without) compared to control.

Treatment	Percentage (%) Difference with/without NO Application (with–without) Compared to Control
	RWC
25 mM	−1.0 ns
50 mM	−1.8 ns
75 mM	−1.8 ns
100 mM	−1.1 ns

RWC: relative water content; ns: not significant.

**Table 8:** Increase in photosynthetic pigments with NO application.

Treatment	Percentage (%) Increase with NO Application			
	*Chl a*	*Chl b*	*Total Chl*	Ct
Control	21.4 *	0.02 ns	15.4 *	2.1 ns
25 mM	15.4 *	3.5 ns	8.3 *	7.7 *
50 mM	40.0 *	15.7 *	19.0 *	28.1 *
75 mM	37.8 *	11.1 *	35.3 *	80.0 *
100 mM	93.3 *	12.5 *	50.0 *	66.7 *

*Chl a*: chlorophyll a; *Chl b*: chlorophyll b; *Total Chl*: total chlorophyll; Ct: carotenoids; ns: not significant; *: significant at *p* < 0.01.

**Table 9:** Decrease in photosynthetic pigments in salinity treatments without NO application compared to control.

Treatment	Percentage (%) Decrease without NO Application Compared to Control			
	*Chl a*	*Chl b*	*Total Chl*	Ct
25 mM	7.1 *	14.3 *	6.9 *	17.4 *
50 mM	21.4 *	19.0 *	19.2 *	30.4 *
75 mM	35.7 *	31.7 *	34.6 *	56.5 *
100 mM	54.3 *	36.5 *	46.1 *	69.6 *

*Chl a*: chlorophyll a; *Chl b*: chlorophyll b; *Total Chl*: total chlorophyll; Ct: carotenoids; *: significant at *p* < 0.01.

**Table 10:** Decrease in photosynthetic pigments in salinity treatments with NO application compared to control.

Treatment	Percentage (%) Decrease with NO Application Compared to Control			
	*Chl a*	*Chl b*	*Total Chl*	Ct
25 mM	6.5 *	7.8 *	6.7 *	10.6 *
50 mM	20.7 *	12.5 *	18.7 *	14.9 *
75 mM	25.6 *	20.3 *	23.3 *	21.3 *
100 mM	28.0 *	29.7 *	26.7 *	48.9 *

*Chl a*: chlorophyll a; *Chl b*: chlorophyll b; *Total Chl*: total chlorophyll; Ct: carotenoids; *: significant at *p* < 0.01.

**Table 11:** Difference in photosynthetic pigments in salinity treatments with/without NO application (with–without) compared to control.

Treatment	Percentage (%) Difference with/without NO Application (with–without) Compared to Control			
	*Chl a*	*Chl b*	*Total Chl*	Ct
25 mM	−0.6 ns	−6.5 *	−0.2 ns	−6.8 *
50 mM	−0.7 ns	−6.5 *	−0.5 ns	−15.5 *
75 mM	−10.1 *	−11.4 *	−11.3 *	−35.2 *
100 mM	−26.3 *	−6.8 *	−19.4 *	−20.7 *

*Chl a*: chlorophyll a; *Chl b*: chlorophyll b; *Total Chl*: total chlorophyll; Ct: carotenoids; −: decrease; ns: not significant; *: significant at *p* < 0.01.

**Table 12:** Increase in ascorbic acid content with NO application.

Treatment	Percentage (%) Increase with NO Application
	AA
Control	4.0 *
25 mM	9.1 *
50 mM	12.2 *
75 mM	13.9 *
100 mM	21.9 *

AA: ascorbic acid; *: significant at *p* < 0.01.

**Table 13:** Decrease in ascorbic acid content in salinity treatments with/without NO application compared to control.

Treatment	Percentage (%) Decrease with/without NO Application Compared to Control	
	AA (without NO Application)	AA (with NO Application)
25 mM	12.0 *	6.7 *
50 mM	18.0 *	10.5 *
75 mM	28.0 *	20.0 *
100 mM	36.0 *	23.8 *

AA: ascorbic acid; *: significant at *p* < 0.01.

**Table 14:** Difference in ascorbic acid content with/without NO application (with–without) compared to Control.

Treatment	Percentage (%) Difference with/without NO Application (with–without) Compared to Control
	AA
25 mM	−5.3 *
50 mM	−7.5 *
75 mM	−8.0 *
100 mM	−12.2 *

AA: ascorbic acid; −: decrease; *: significant at *p* < 0.01.

**Table 15:** Increase in oxidative compound activity with NO application.

Treatment	Percentage (%) Increase with NO Application			
	SOD	CAT	POD	APX
Control	2.7 ns	22.2 *	18.2 *	8.6 *
25 mM	8.9 *	8.7 *	25.0 *	16.7 *
50 mM	16.3 *	11.1 *	21.4 *	6.3 *
75 mM	7.1 *	15.4 *	11.8 *	9.4 *
100 mM	10.3 *	9.7 *	14.3 *	19.0 *

SOD: superoxide dismutase; CAT: catalase; POD: peroxidase; APX: ascorbate peroxidase; ns: not significant; *: significant at *p* < 0.01.

**Table 16:** Increase in oxidative compound activity in salinity treatments without NO application compared to control.

Treatment	Percentage (%) Increase without NO Application Compared to Control			
	SOD	CAT	POD	APX
25 mM	25.0 *	27.8 *	9.1 *	16.7 *
50 mM	36.1 *	38.9 *	27.3 *	34.7 *
75 mM	50.0 *	50.0 *	54.5 *	43.1 *
100 mM	61.1 *	72.2 *	90.9 *	65.3 *

SOD: superoxide dismutase; CAT: catalase; POD: peroxidase; APX: ascorbate peroxidase; *: significant at *p* < 0.01.

**Table 17:** Increase in oxidative compound activity in salinity treatments with NO application compared to control.

Treatment	Percentage (%) Increase with NO Application Compared to Control			
	SOD	CAT	POD	APX
25 mM	28.9 *	18.2 *	15.4 *	28.9 *
50 mM	50.0 *	27.3 *	30.8 *	34.9 *
75 mM	57.9 *	31.8 *	56.1 *	55.3 *
100 mM	68.4 *	54.5 *	94.6 *	84.2 *

SOD: superoxide dismutase; CAT: catalase; POD: peroxidase; APX: ascorbate peroxidase; *: significant at *p* < 0.01.

**Table 18:** Difference in oxidative compound activity in salinity treatments with/without NO application (with–without) compared to control.

Treatment	Percentage (%) Difference with/without NO Application (with–without) Compared to Control			
	SOD	CAT	POD	APX
25 mM	+3.9 *	−9.6 *	+6.3 *	+12.2 *
50 mM	+13.9 *	−11.6 *	+3.5 *	+0.2 ns
75 mM	+7.9 *	−18.2 *	+1.6 ns	+12.2 *
100 mM	+7.3 *	−17.7 *	+3.7 *	+18.9 *

SOD: superoxide dismutase; CAT: catalase; POD: peroxidase; APX: ascorbate peroxidase; +: increase; −: decrease; ns: not significant; *: significant at *p* < 0.01.

**Table 19:** Variation in non-oxidative compound content with NO application.

Treatment	Percentage (%) Variation (Increase/Decrease) with NO Application			
	Pro	MDA	TP	H_2_O_2_
Control	+25.0 *	+50.0 *	+7.5 *	+8.3 ns
25 mM	+27.8 *	+20.5 *	+14.3 *	−12.5 *
50 mM	+18.2 *	+28.9 *	+19.4 *	−28.6 *
75 mM	+7.7 *	+25.0 *	+24.1 *	−31.2 *
100 mM	+9.4 *	+26.8 *	+20.0 *	−29.7 *

Pro: proline; MDA: malondialdehyde; H_2_O_2_: hydrogen peroxide; +: increase; −: decrease; ns: not significant; *: significant at *p* < 0.01.

**Table 20:** Variation in non-oxidative compound content in salinity treatments without NO application compared to control.

Treatment	Percentage (%) Variation (Increase/Decrease) without NO Application Compared to Control			
	Pro	MDA	TP	H_2_O_2_
25 mM	+25.0 *	+35.7 *	−10.0 *	+54.5 *
50 mM	+37.5 *	+60.7 *	−22.5 *	+154.5 *
75 mM	+62.5 *	+61.1 *	−25.0 *	+190.9 *
100 mM	+100.0 *	+153.6 *	−37.5 *	+236.4 *

Pro: proline; MDA: malondialdehyde; H_2_O_2_: hydrogen peroxide; +: increase; −: decrease; ns: not significant; *: significant at *p* < 0.01.

**Table 21:** Variation in non-oxidative compound content in salinity treatments with NO application compared to control.

Treatment	Percentage (%) Variation (Increase/Decrease) with NO Application Compared to Control			
	Pro	MDA	TP	H_2_O_2_
25 mM	+14.3 *	+11.9 *	−5.9 *	+25.0 *
50 mM	+23.8 *	+40.5 *	−12.9 *	+66.7 *
75 mM	+38.9 *	+60.7 *	−17.6 *	+83.3 *
100 mM	+71.4 *	+114.3 *	−29.4 *	+108.3 *

Pro: proline; MDA: malondialdehyde; H_2_O_2_: hydrogen peroxide; +: increase; −: decrease; ns: not significant; *: significant at *p* < 0.01.

**Table 22:** Difference in non-oxidative compound content in salinity treatments with/without NO application (with–without) compared to control.

Treatment	Percentage (%) Difference with/without NO Application (with–without) Compared to Control			
	Pro	MDA	TP	H_2_O_2_
25 mM	−10.7 *	−23.8 *	−4.1 *	−29.5 *
50 mM	−13.7 *	−20.2 *	−9.6 *	−87.8 *
75 mM	−23.6 *	−0.4 ns	−7.4 *	−107.6
100 mM	−28.6 *	−39.3 *	−8.1 *	−128.1 *

Pro: proline; MDA: malondialdehyde; H_2_O_2_: hydrogen peroxide; −: decrease; ns: not significant; *: significant at *p* < 0.01.

**Table 23:** Increase in leaf and root nitrogen and phosphorus contents with NO application.

Treatment	Percentage (%) Increase with NO Application			
	Leaf N	Root N	Leaf P	Root P
Control	9.7 *	26.7 *	5.7 *	27.8 *
25 mM	3.4 ns	17.6 ns	3.0 ns	29.4 ns
50 mM	3.7 ns	28.0 ns	9.7 *	33.3 *
75 mM	3.8 ns	21.7 ns	14.8 *	45.4 *
100 mM	4.0 ns	25.0 *	15.4 *	50.0 *

ns: not significant; *: significant at *p* < 0.01.

**Table 24:** Decrease in leaf and root nitrogen and phosphorus contents in salinity treatments without NO application compared to control.

Treatment	Percentage (%) Decrease without NO Application Compared to Control			
	Leaf N	Root N	Leaf P	Root P
25 mM	8.2 *	26.7 *	5.7 ns	11.1 ns
50 mM	14.6 *	40.0 *	14.3 *	16.7 *
75 mM	17.7 *	48.9 *	22.9 *	33.3 *
100 mM	20.9 *	55.5 *	25.7 *	44.4 *

ns: not significant; *: significant at *p* < 0.01.

**Table 25:** Decrease in leaf and root nitrogen and phosphorus contents in salinity treatments with NO application compared to control.

Treatment	Percentage (%) Decrease with NO Application Compared to Control			
	Leaf N	Root N	Leaf P	Root P
25 mM	11.8 *	27.6 *	5.4 *	8.7 ns
50 mM	17.6 *	46.5 *	13.5 *	13.0 *
75 mM	22.3 *	50.0 *	18.9 *	30.4 *
100 mM	23.5 *	56.9 *	18.9 *	34.8 *

ns: not significant; *: significant at *p* < 0.01.

**Table 26:** Difference in leaf and root nitrogen and phosphorus contents in salinity treatments with/without NO application (with–without) compared to control.

Treatment	Percentage (%) Difference with/without NO Application (with–without) Compared to Control			
	Leaf N	Root N	Leaf P	Root P
25 mM	+3.6 *	+0.9 ns	−0.3 ns	−2.4 *
50 mM	+3.0 *	+6.5 *	−0.8 ns	−3.7 *
75 mM	+4.6 *	+1.1 ns	−4.0 *	−2.9 *
100 mM	+3.6 *	+1.4 ns	−6.8 *	−9.6 *

+: increase; −: decrease; ns: not significant; *: significant at *p* < 0.01.

**Table 27:** Variation in leaf and root K^+^ and Na^+^ contents with NO application.

Treatment	Percentage (%) Variation (Increase/Decrease) with NO Application			
	Leaf K^+^	Leaf Na^+^	Root K^+^	Root Na^+^
Control	+12.2 *	−8.3 ns	+4.3 *	−4.3 ns
25 mM	+25.0 *	−11.8 *	+9.5 *	−5.3 ns
50 mM	+26.1 *	−18.2 *	+21.0 *	−9.1 *
75 mM	+13.0 *	−19.2 *	+22.2 *	−8.7 *
100 mM	+6.7 *	−22.6 *	+17.6 *	−18.5 *

+: increase; −: decrease; ns: not significant; *: significant at *p* < 0.01.

**Table 28:** Variation in leaf and root K^+^ and Na^+^ contents in salinity treatments without NO application compared to control.

Treatment	Percentage (%) Variation (Increase/Decrease) without NO Application Compared to Control			
	Leaf K^+^	Leaf Na^+^	Root K^+^	Root Na^+^
25 mM	−6.1 ns	+41.7 *	−8.7 *	+22.0 *
50 mM	−4.1 ns	+83.3 *	−17.4 *	+46.7 *
75 mM	−4.1 ns	+116.7 *	−23.9 *	+53.3 *
100 mM	−7.1 *	+166.7 *	−30.4 *	+73.3 *

+: increase; −: decrease; ns: not significant; *: significant at *p* < 0.01.

**Table 29:** Variation in leaf and root K^+^ and Na^+^ contents in salinity treatments with NO application compared to control.

Treatment	Percentage (%) Variation (Increase/Decrease) with NO Application Compared to Control			
	Leaf K^+^	Leaf Na^+^	Root K^+^	Root Na^+^
25 mM	+9.1 *	+36.4 *	−4.2 *	+21.4 *
50 mM	+5.5 *	+63.6 *	−2.1 *	+35.7 *
75 mM	−3.6 ns	+90.9 *	−8.3 *	+50.0 *
100 mM	−9.1 *	+118.2 *	−20.8 *	+57.1 *

+: increase; −: decrease; ns: not significant; *: significant at *p* < 0.01.

**Table 30:** Difference in leaf and root K^+^ and Na^+^ contents in salinity treatments with/without NO application (with–without) compared to control.

Treatment	Percentage (%) Difference with/without NO Application (with–without) Compared to Control			
	Leaf K^+^	Leaf Na^+^	Root K^+^	Root Na^+^
25 mM	+3.0 *	−5.3 *	−4.5 *	−0.6 ns
50 mM	+1.4 ns	−19.7 *	−15.3 *	−11.0 *
75 mM	−0.5 ns	−25.8 *	−15.6 *	−3.3 *
100 mM	+2.0 *	−48.5 *	−9.6 *	−16.2 *

+: increase; −: decrease; ns: not significant; *: significant at *p* < 0.01.

## Data Availability

All generated data are enclosed within the manuscript.
